# Biting the Finger Nails

**Published:** 1888-12

**Authors:** 


BITING THE FINGER NAILS.—" A novel incident resulting from a habit of very common prevalence among nervous people was brought to my notice recently," said a leading physician of Philadelphia, the other day. " A young lady presented herself at my office and complained of a constant irritation in her throat. Two weeks previously she had been taken with a very severe attack of sore throat, which was treated by the family physician. Under his care, she said, the inflammation quickly subsided, but there still remained a sensation of irritation. Examination revealed a small, fleshy looking object the size of a kernel of wheat adherent to the tissues posterior to the left tonsil by the one end. The other parts of the throat were normal. The little mass could not be detached by a cotton-covered probe, but by the use of forceps it was easily removed, and, on examination, proved to be a piece of finger nail which had becone imbedded in a cheesy deposit. A broken piece of the nail was also removed from under the mucous membrane at the same spot by a sharp pointed probe. The lady then confessed to the habit of biting her finger nails, and moreover could remember that a day or two previous to her throat trouble a piece of nail she had bitten off had become lost in her mouth, but, after it had caused a fit of coughing, she had forgotten all about it until reminded by the discovery."

